# A Century of the Evolution of the Urban Area in Shenyang, China

**DOI:** 10.1371/journal.pone.0098847

**Published:** 2014-06-03

**Authors:** Miao Liu, Yanyan Xu, Yuanman Hu, Chunlin Li, Fengyun Sun, Tan Chen

**Affiliations:** 1 State Key Laboratory of Forest and Soil Ecology, Institute of Applied Ecology, Chinese Academy of Sciences, Shenyang, China; 2 University of Chinese Academy of Sciences, Beijing, China; NASA Jet Propulsion Laboratory, United States of America

## Abstract

Analyzing spatiotemporal characteristics of the historical urbanization process is essential in understanding the dynamics of urbanization and scientifically planned urban development. Based on historical urban area maps and remote sensing images, this study examined the urban expansion of Shenyang from 1910 to 2010 using area statistics, typology identification, and landscape metrics approaches. The population and gross domestic product were analyzed as driving factors. The results showed that the urban area of Shenyang increased 43.39-fold during the study period and that the growth rate has accelerated since the 1980s. Three urban growth types were distinguished: infilling, edge-expansion, and spontaneous growth. Edge-expansion was the primary growth type. Infilling growth became the main growth type in the periods 1946–70, 1988–97, and 2004–10. Spontaneous growth was concentrated in the period of 1997 to 2000. The results of landscape metrics indicate that the urban landscape of Shenyang originally was highly aggregated, but has become increasingly fragmented. The urban fringe area was the traditional hot zone of urbanization. Shenyang was mainly located north of the Hun River before 1980; however, the south side of the river has been the hot zone of urbanization since the 1980s. The increase of urban area strongly correlated with the growth of GDP and population. Over a long time scale, the urbanization process has been affected by major historical events.

## Introduction

Nearly 50% of the human population (3.3 billion) lived in urban areas in 2008, and this number is expected to reach 5 billion by 2030 [1]. The world is currently undergoing an unprecedented process of urbanization [2]. The urban area plays an important role in the regional economy as the spatial unit where most economic activities occur. Both the scale and rate of this urban expansion are extraordinary. The first urban transition took place in Europe and North America from 1750 to 1950, when the urban population of these places increased from 15 million to 423 million. The second urban transition (1950–2030) is happening largely in Africa and Asia, and it will increase their urban population from 309 million to 3.9 billion in only 80 years [3], [4].

The conversion of rural lands to urban or other built-up uses is the most drastic form of land-use change [5]–[7], and it is a key research topic in landscape ecology [8], [9]. Although urbanized areas cover only about 3% of the earth's land surface, they account for more than 78% of carbon emissions, 60% of residential water use, and 76% of the wood used for industrial purposes [10]. The influence of urbanized areas on biodiversity and ecosystem functioning and services extends far beyond the limits of cities [11], [12]. To understand the process of urbanization as well as its ecological consequences, it is necessary to quantify the spatiotemporal patterns of urbanization [13].

Urban patterns and dynamics have been extensively studied over the past century. Many classical urban theories have been developed, such as the concentric zone theory [14], sector theory [15], multiple nuclei theory [16], catastrophe theory [17]. In the past several decades, some theories were applied in urban studies, such as chaos theory [18], dissipative structure theory [19], percolation theory [20], self-organization theory [21], and fractal theory [22]. With the development of computers, geographic information systems (GIS), and remote sensing (RS), new technologies and methods were used in the spatial analysis and simulation of urbanization, such as with non-equilibrium and non-linear system perspectives [23], models and forecasting patterns of urban systems, cellular automata [24], [25], agent-based simulation [26], and the entropy method [27]. These methods and models have provided a deeper understanding of urban structure and dynamics.

RS is a significant data source for urban analysis, offering high spatial and temporal accuracy and consistency [28]. With development of commercial satellites since the 1970s, a number of remote sensing data types can be accessed, such as Landsat, Spot, Quickbird and so on. These data present useful sources for studying urban dynamics and improving the modeling of urban systems [29]. Over recent decades, various approaches for urban land-use classification and change detection have been developed to facilitate urban analysis based on RS data [30]–[32]. Historical geographic data and maps, such as aerial photographs and cartographic maps, enable the evaluation of land use change before RS application. The old maps were usually processed and analyzed through digitization with GIS. Numerous authors have studied ways of using these maps [33]–[35].

The urbanization process is a consequence of the interaction of various kinds of driving forces, including natural and socioeconomic factors [36]. The spatial heterogeneity of these factors causes different typologies of urban sprawl. The study of driving factors of land-use change is one of the main research topics of landscape ecology [7], and is also relevant for ecology [37].

Some studies have focused on the urban structure and fractal distribution, especially with Fractal geometry [38]–[41]. Camagni et al. (2002) distinguished five types of urban growth: infilling, expansion, linear development, sprawl, and large-scale projects. Wilson et al. (2002) identified five types of urban growth: infill, expansion, isolated, linear branch, and clustered branch. Basically, three main types of urban growth have been documented: infilling, edge-expansion, and spontaneous growth [12]. Infilling signifies a non-urban area surrounded by an urban area being converted to urban land; edge-expansion, also called urban fringe development, refers to a newly developed urban area spreading out from the fringe of an existing urban area; and spontaneous growth means that a new urban area is formed without direct spatial connection to an existing urban area.

In the last few decades, landscape metrics were developed with landscape ecology and GIS technology. Landscape pattern metrics are useful methods for quantifying spatial patterns, which have been wildly employed in landscape pattern analysis and urban landscape studies [32], [42], [43]. Many studies have shown the availability of landscape metrics for describing the temporal dynamics of urban landscapes [13], [23], [44]. Large numbers of landscape metrics were developed in the last few decades [45]. The validity of landscape metrics, response to scaling change and relevance among metrics were analyzed [8], [46], providing the basis of choosing landscape metrics for different purposes.

Shenyang is the central and largest city in northeast China. Some cases studied the urban expansion [47], [48] and land use change [49] in the last several decades based on remote sensing images. Wu et al. (2009) predicted the future urban expansion from 2005 to 2030 with the SLEUTH model [50]. Most data in these studies were derived from RS images. The temporal scale mainly began after RS images became available (Landsat MSS and TM images since 1972).The main purpose of the present study was to quantify historical urban land use changes and driving factors during the last century in Shenyang, using typology of urban growth and landscape metrics methods. Through this long-time urbanization process study, we aim to explore the characters of urban spatial expansion in China.

## Methods

### Study area

**Figure 1:**
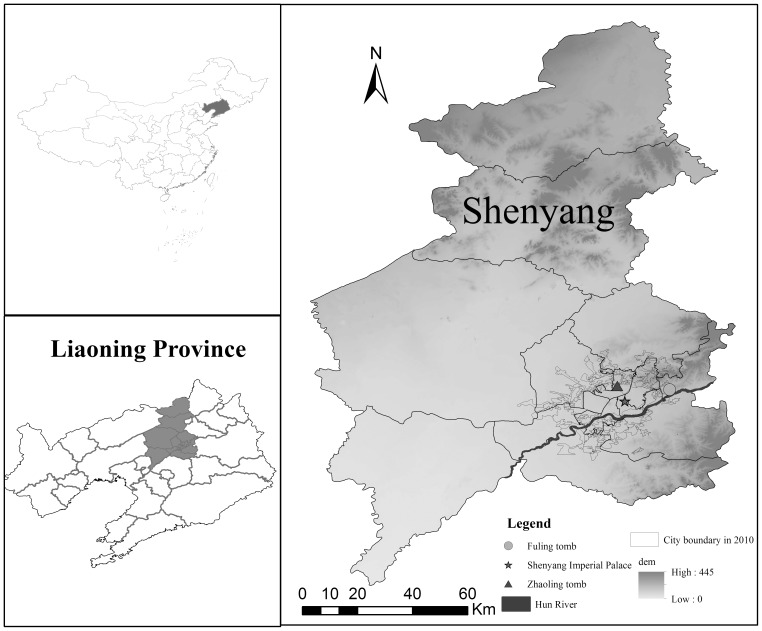
Location of the study area.

The city of Shenyang (41°11′–42°02′N; 122°25′–123°48′E) lies in the transition zone between a branch of the Changbai Mountains and the flood plain of the Liao River in Northeast China (Fig. 1). The major topography is characterized by an alluvial plain in the west, with low hilly lands in the northeast and southeast. Shenyang is the capital of Liaoning Province, and it is the communication, commercial, scientific, and cultural centre of Northeast China. Its annual average temperature is 7.8°C and its annual precipitation is 707 mm (1906–2002). As a key investment and industrial base designated by the Chinese government since 1948, Shenyang developed into the centre of Chinese heavy industrial development before the 1980s. With the growth of its population and industry, Shenyang has continuously expanded its borders.

### Data collection

In this study, many spatial data sets were collected, including multi-temporal historical city maps, and a time series of Landsat Thematic Mapper data (Table 1). All the historic city maps include the information of build-up area and main roads. The city boundary from 1910 to 1970was interpreted from the downtown area, which had clear boundary. The image-to-image method was used for the geo-referenced registration of other images with a total root mean square error of less than 0.5 pixels (cell size 30 m). The historical maps did not have a coordinate system before 1980. The relief map for 1980 was geometrically calibrated with the surveying maps, which were produced by the Chinese Bureau of Land and Resources in 1979. All maps in other years were calibrated based on the map in 1980. Three landmark buildings with a long history and unchanged locations in Shenyang were employed as reference points: Zhaoling tomb, the Qing dynasty imperial mausoleum, located in the north side was built in 1651; Fuling tomb, another Qing dynasty imperial mausoleum which was built in 1629; and the Shenyang Imperial Palace, which was completed in 1636 (Fig.1). Main roads around these reference points have not changed since the Shenyang city was built. Historical maps before 1980 were geometrically corrected based on the three landmark buildings and the main roads around them.

**Table 1:** List of spatial dataset used in the study.

No	Dataset	Time (Year)	Source	Producer	Original resolution or scale
1	Elevation	1981	Chinese second-generation 1∶100,000 relief maps	Liaoning surveying and mapping bureau	25 m
2	TM and ETM images	1988, 1992, 1997, 2000, 2010	USGS (United States Geological Survey)	USGS (United States Geological Survey)	30 m
3	Aerial images	2004	Liaoning surveying and mapping bureau	Liaoning surveying and mapping bureau	10 m
4	Shenyang city map	1910,	Shenyang Urban Construction Archives	Surveying and mapping agency of Qing dynasty	1∶100,000
5		1920,	Shenyang Urban Construction Archives	Surveying and mapping agency of local government	1∶100,000
6		1931, 1939,	Shenyang Urban Construction Archives	Surveying and mapping agency of puppet Manchurian regime	1∶100,000
7		1946, 1961, 1970	Shenyang Urban Construction Archives	Liaoning surveying and mapping bureau	1∶50,000

Visual interpretation (using local knowledge) from Landsat TM images and aerial images was carried out to form a binary map of urban/non-urban classes. In interpretation, we classified the urban area based on built-up areas surrounding the downtown region. To determine the accuracy of the image classification, the stratified random sampling method (Jensen 1996) was used to generate 96 reference points for each of the classified images. 140 reference points were located in the field with the help of a global positioning system (GPS) with ±5 m error for ground-truthing in 2010. The attributes of each point from 1981 to 2010 were collected through field survey with local residents. The kappa accuracy index (Congalton 1991) was 85.5% in 1981, 87.6% in 1988, 88.3% in 1992, 89.1% in 1997, 90.2% in 2000, 95.8% in 2004 and 92.2% in 2010. The accuracy of urban maps compared to historical maps was not measured owing to the absence before 1980 of coordinate systems. All the maps were converted to raster data in ARCGIS with spatial resolution 30 m, which is the pixel size of Landsat images.

Except for the spatial data, the statistical books of Shenyang in 1979, 1985, 1990, 2005 and 2010 were used to collect social-economic data.

The images from 1910 to 1970 were urban area maps, which have a clean city boundary. The maps of the urban area from 1988 to 2010 were interpreted based on remote sensing images. The boundary of the urban area was delineated based on the emergence of new urban patches at the edge of the urban area in each previous period.

### Typology of urban growth

**Figure 2:**
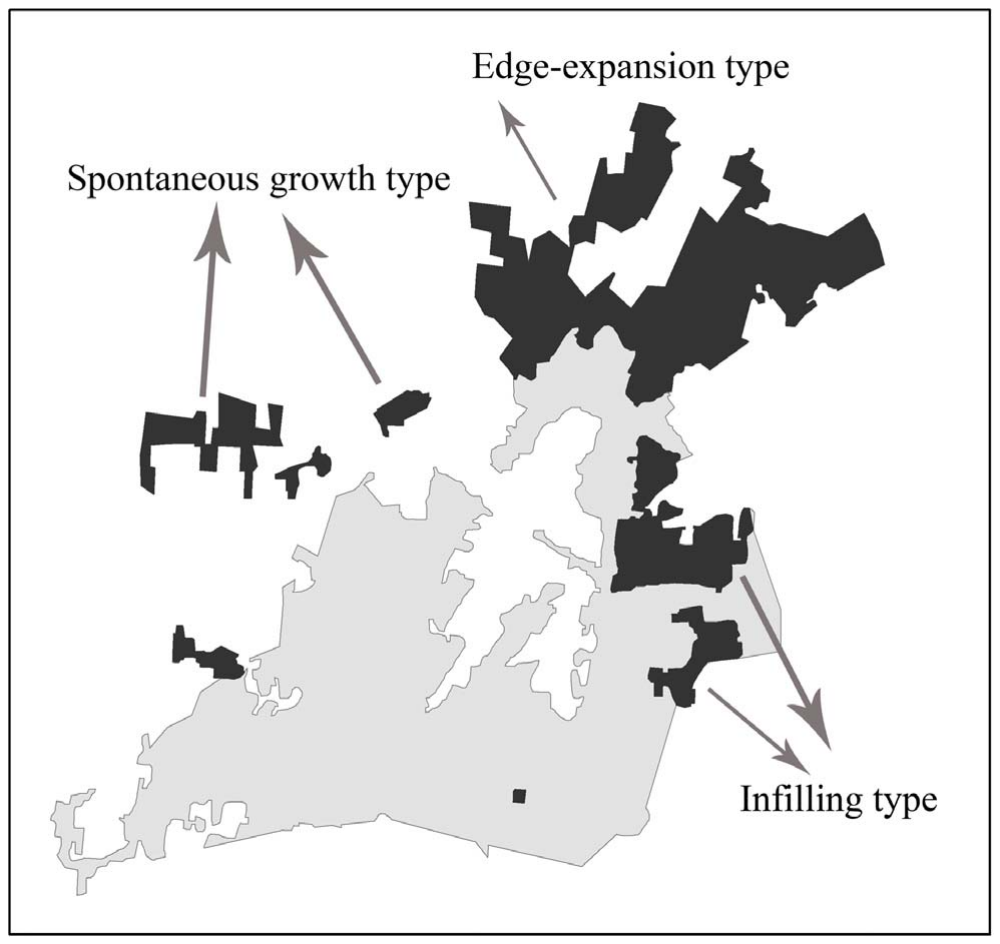
Typology of urban growth. The grey area represents the pre-growth urban patches and the dark area represents the newly grown urban patches.

A simple quantitative method was used to distinguish the three growth types (Fig.2) using the following equation: 



Where Lc is the length of the common boundary between a newly grown urban area and pre-growth urban patches, and P is the perimeter of this newly grown area. The urban growth type was identified as follows: infilling when S≥0.5; edge-expansion when 0<S<0.5; and spontaneous growth when S = 0, which indicates no common boundary. Xu et al. (2005) proved the effectiveness of this approach.

### Landscape metrics

To characterize urban landscape pattern effectively and succinctly, four metrics were chosen to reflect urban patch characters and spatial distribution based on previous studies of the meaning and representativeness of metrics [12], [13], [46], [51], including number of patches (NP), Patch density (PD), landscape shape index (LSI), and aggregation index (AI) (Table 2). These four metrics can reflect the urban expansion landscape pattern comprehensively from the patch class level. The landscape metrics were calculated using FRAGSTATS 4.1 software [52].

**Table 2:** Landscape metrics used to quantify spatial pattern.

Acronym	Scale	Index name	Description
NP	Class or landscape	number of patches	Number of patches for each class
PD	Class or landscape	patch density	Quantifies the number of patches of the corresponding patch type divided by total landscape area (m^2^).
LSI	Class or landscape	landscape shape index	A modified perimeter-area ratio of the form that measures the shape complexity of the whole landscape or a specific patch type.
AI	Class or landscape	aggregation index	Shows the frequency with which different pairs of patch types appear side-by-side on the map

## Results

**Figure 3:**
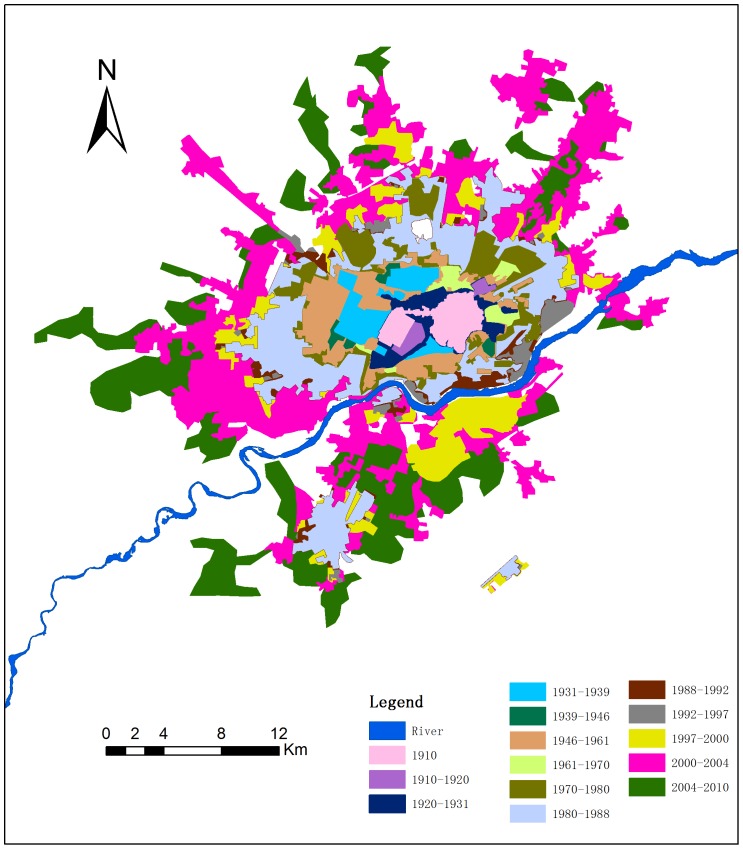
Urban spatial expansion of Shenyang from 1910 to 2010. (Buffer distance from the Shenyang Imperial Palace: 0–15 kilometers step 1kilometer; 15–25 kilometers step 5 kilometers.).

As the Fig.3 show, the Hun River flows through Shenyang, and the old urban area was located north of the river in 1910. Expanded urban patches were also concentrated north of the Hun River before 1980. Since the 1980s, large urban area patches appeared south of the river. Between 1980 and 2004, more than half of the new urban patches still were located north of the Hun River. Since 2004, more than half of the new patches have been located south of the river.

**Figure 4:**
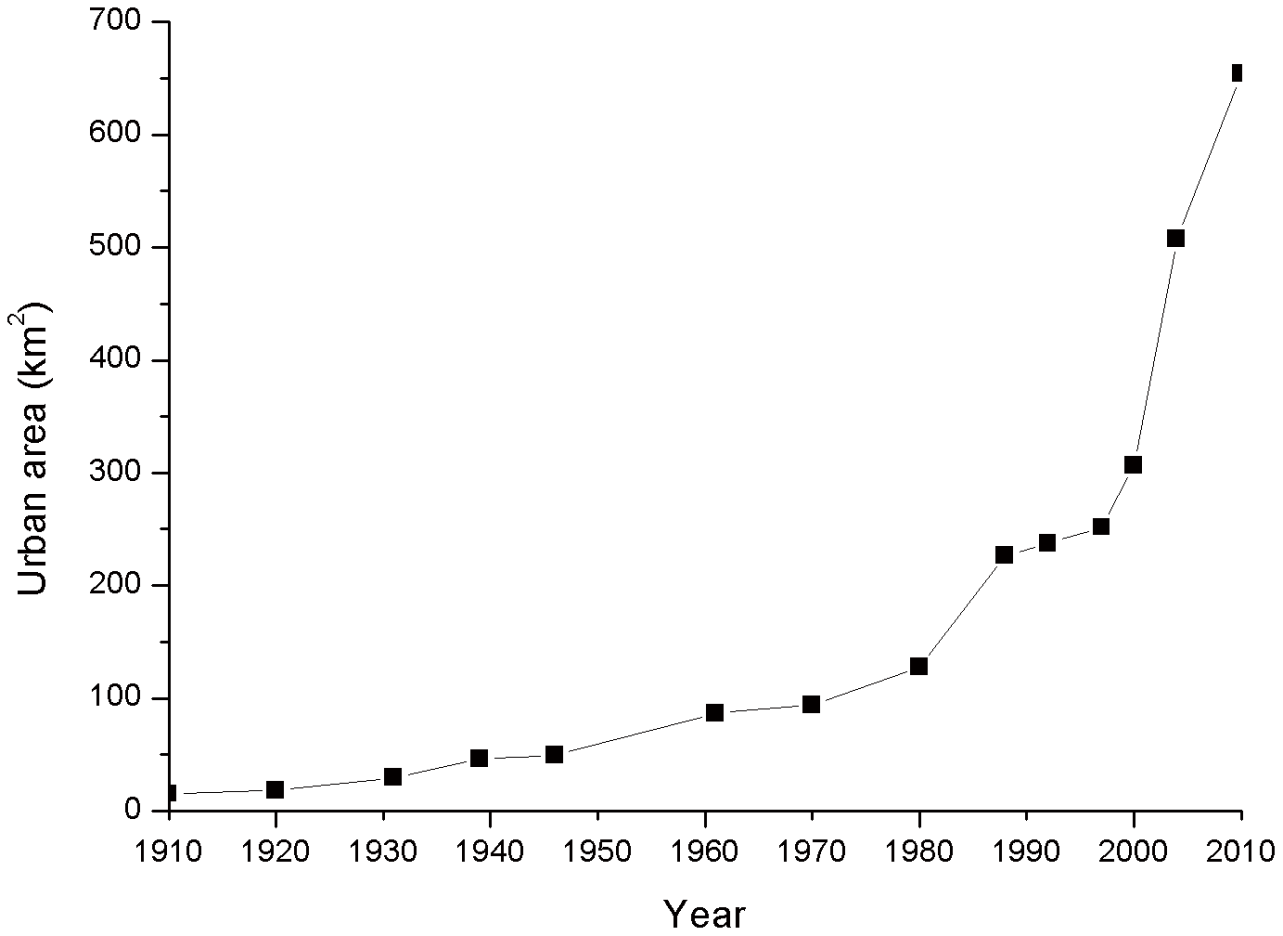
Urban area change of Shenyang from 1910 to 2010.

Over the last century (1910–2010), the urban area of Shenyang has shown a continuous increase (Fig.4). The urbanized area was 15.07 km^2^ in 1910 and 653.91 km^2^ in 2010—a 43.39-fold increase. The mean annual growth rate of urban area was 1.61 km^2^/year from 1910 to 1980, 7.29 km^2^/year from 1980 to 1997, and 17.54 km^2^/year from 1997 to 2010. The urban area growth rate and urban population has significantly increased since the 1980s, which indicates that the urbanization process of Shenyang has been accelerating.

**Figure 5:**
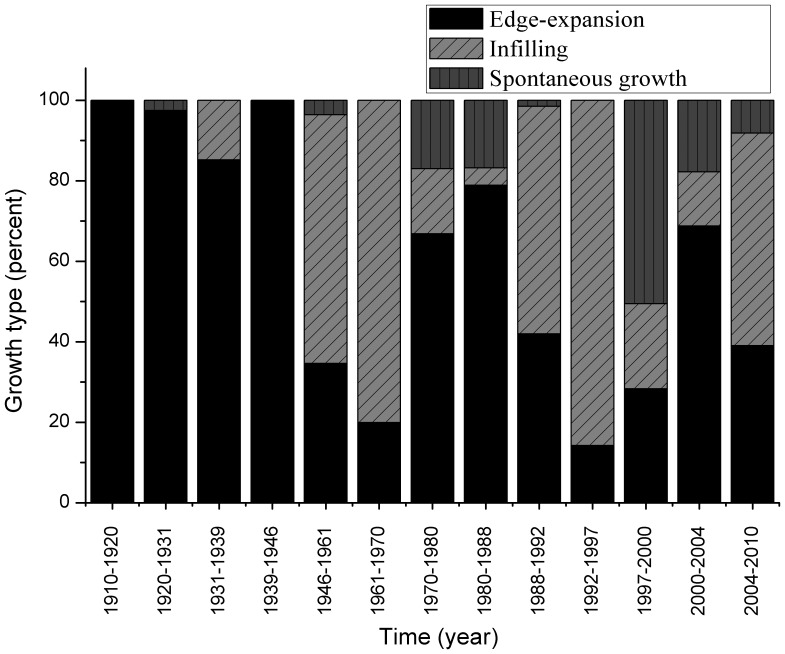
Proportion of the three growth types in different periods.

The contribution of the three urban area growth types to the increased area is presented in Fig.5. Over the 100 years of the study period, the area of edge-expansion, infilling, and spontaneous growth was 775.40, 406.83, and 117.77 ha respectively. Edge-expansion was the primary growth type, accounting for 59.65%. Before 1946, the growth areas were mostly of the edge-expansion type. In the periods 1970–88 and 2000–04, edge-expansion accounted for over 70% of the growth. However, infilling became the main growth type during the periods 1946–70, 1988–97, and 2004–10. In contrast, spontaneous growth accounted only for 9.06% of the growth over the past century, and it was concentrated in the period of 1997 to 2000.

**Figure 6:**
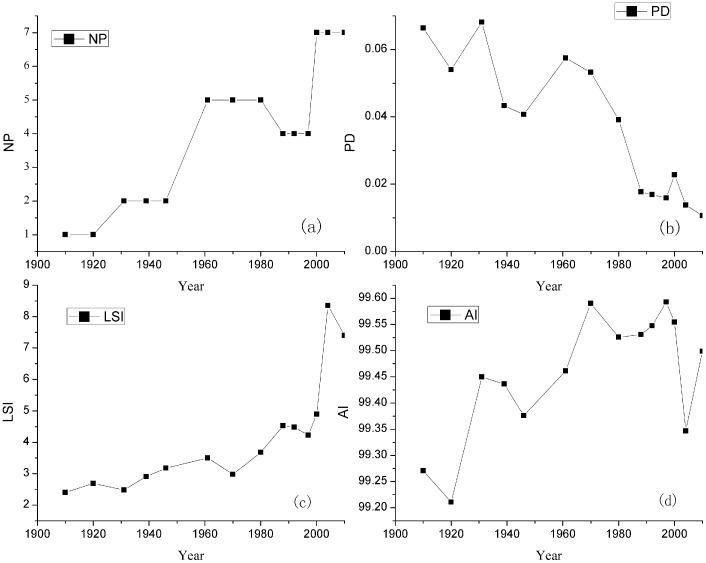
Change in the landscape indices during the period 1910–2010: (a) number of patches (NP), (b)Patch density (PD), (c) landscape shape index (LSI), and (d) aggregation index (AI).

The trajectories of landscape metrics are shown in Fig.6. NP increased with the emergence of the spontaneous growth type (Fig.6(a)). NP showed an increasing trend with the urban expansion. However, NP decreased in the 1990s because the open space in the urban patches showed infilling growth. PD quantifies the density of patches, which kept decreasing in the study period (Fig.6(b)). The decrease of PD indicates that the mean scale of patches kept increasing and single urban patches became larger. LSI qualifies the shape complexity of patches (Fig.6(c)). LSI maintained an increasing trend, which indicates that the patch shapes became more and more complicated. LSI had a dramatic increase from 2000 to 2004, which was associated with fast development. The value of AI was nearly 100 from 1910 to 2010, which indicates that urban patches show a very high degree of concentrated spatial distribution (Fig.6(c)).

### Driving factor analysis

**Figure 7:**
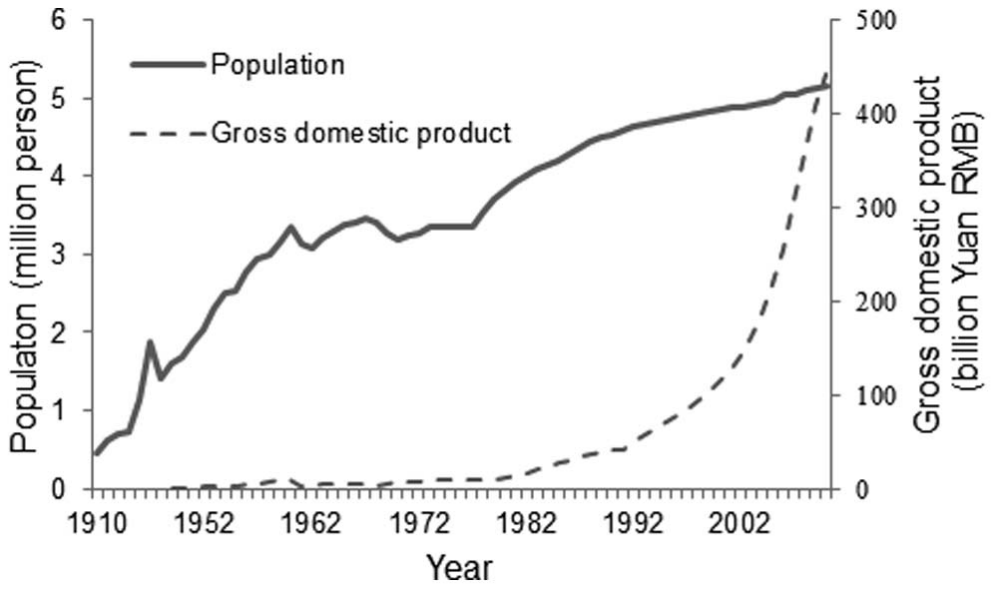
The gross domestic product and population of Shenyang from 1919 to 2010.

Driving factors are the forces that cause observed land use changes, i.e., they are influential processes in the evolutionary trajectory of the land use. Urban expansion and land use change are forced by geographical and social-economical factors. Urban expansion and related land use change is decided by a combination of these factors. Shenyang city locates in the flood plain of the Liao River. The relief of study area is smooth (elevation ranges from 58 to 124 m), so the geographical factors, such as elevation, slope, aspect, do not influence or limit urban expansion. The population of Shenyang city from 1910 to 2010 was available (Fig.7). However, due to the absence of data before 1949, gross domestic product (GDP) of Shenyang city only was available from 1949 to 2010 The GDP data were converted to the comparative GDP in 1952.

The increase of urban area strongly correlates with the growth of GDP and population in a linear form (*r* ^2^ = 0.93 and 0.86 correspondingly). The GDP correlation demonstrates the economy was the more dominant factor for urban expansion than population in Shenyang from 1910 to 2010.

## Discussion

### Shift in the growth hot zone

Urbanization is driven by natural and cultural factors. Analyzing the shift in the urban growth hot zone has some useful implications for urban planning and modeling [24]. All the new-growth urban areas before 1980 in Shenyang occurred in the zones around the pre-growth urban patches before 1980, which are located north of the Hun River. Over 90% of the total growth took place in zones within a distance of 3 km from the edge of the pre-growth urban patches. The buffer zone of 0–3 km of built-up urban area, in which the Shenyang Imperial Palace north of the Hun River was considered the centre of the city, was the growth hot zone before 1980. Since the 1980s, major spontaneous growth patches have appeared around the urban area. In 1980 to 1988, new urban patches developed in many locations, and new urban patches started appearing south of the Hun River. Many cities in the world developed on both side of a main river, but Shenyang was largely located north of the Hun River before 1980. In traditional Chinese culture, north of the river represents “yang”, which signifies that it is better for living. The name “Shenyang” derives from the city location north of the Hun River, which before 1675 was called the Shen River. With economic development and urban expansion, the urban area of Shenyang has extended south of the Hun River. The buffer zone of 0–4 km of built-up urban area located on either side of the river became a growth hot zone from1980 to 1988. In 1988 to 2000, urban expansion was concentrated in an area south of the Hun River; thus, south of the Hun River became a hot zone. In 2000 to 2010, the urban area showed explosive growth in all directions, with extensive spontaneous patches in the south, north, and west. The explosive urban growth was caused not only by China's economic development but also the local government's land financing in the 2000s. Land financing refers to some local governments relying on the income produced from land-use rights to finance local expenditure. The buffer zone from 0 to 7 km of built-up urban area extending to the east was the growth hot zone from 2000 to 2010. The distance of the hot zone to the built-up urban area has progressively increased, and the new-growth patches have become larger. The speed of urban growth has also become more rapid.

### Major historical events and urbanization

Various kinds of driving forces, including natural and socioeconomic factors, influence the urbanization process [7]. As many studies showed that economic and demographic factors are the main driving forces [53]–[55], these are also the main driving forces in Shenyang urban expansion. However, the effect of national and local historic events may affect in city's long historic expansion. The major events in the long history of Shenyang are listed in Table 3. Shenyang was a small town before the building of its Imperial Palace in 1625. Northeast China was the home region of the Qing dynasty (1636–1911) emperors. The Qing dynasty capital moved to Beijing from Shenyang in 1644. Shenyang was subjected to little development from 1644 to 1911 because as the home of the Qing dynasty, such development was forbidden. After the Xinhai Revolution of 1911, Shenyang was controlled by the Fengtian clique of warlords, and the city began to expand slowly. During the period of the city's occupation by the Japanese (1931–45), railways and factories were built, and new edge-expansion patches appeared in western and northern areas as industrial districts. After the establishment of the People's Republic of China in 1949, Shenyang was positioned as a heavy industrial city. Urban expansion, which was mainly concentrated in the western part, was related to industrial development. The urban area of Shenyang showed a moderate increase from 1949 to 1979. In the first 10 years (1980–90) of reform and opening up, the urban area of Shenyang underwent accelerated growth in line with its economic development. However, numerous factories in Shenyang closed down in and after 1990, which led to substantial job losses and an economic downturn. In 1990–97, urbanization almost came to a halt. Since 1999, the construction of private residential buildings has not been the responsibility of the government, though policies for the construction of commercial residential buildings have been in place. The new accelerated growth in the urban area has mainly been driven by residential construction. To help the heavy industrial cities in Northeast China, policies for reviving previous key industrial bases in that region have been implemented by the central government. Through the integrated effects of these policies and the local government's land financing, the urban area of Shenyang showed an explosive increase. The government of Shenyang city planned to move to south of Hun River. The urban growth hotspot has moved to the area before publishing the plan due to this information of ‘government moving’. The major historical events in the city may not have been the direct driving forces of urbanization, but the economic and political changes brought about by those events have determined the city character of Shenyang and its urbanization. Major historic events have brought a corresponding increase in spontaneous growth (Fig.5). However, the landscape metrics are not sensitive to the major historic events. The reason may be landscape metrics reflect the whole landscape status, but the major historic events mainly affect the new-growth patches.

**Table 3:** Major historical events related to Shenyang.

Year	Historic events
1644	The city was renamed ‘Fengtian’, which was the second capital of Qing Dynasty
1911	The city was capital of the Fengtian clique of warlords after Revolution of 1911
1926	The city was renamed ‘Shenyang’
1931	The city was occupied by Japanese, and renamed ‘Fengtian’
1945	The city was renamed ‘Shenyang’ after the victory of Anti-Japanese War
1949	The People's Republic of China was built
1979	The reform and opening-up police was carried out in China
2003	The police of reviving previous key industrial bases in North-eastern China was carried out
2004	The government of Shenyang plans moving to south of Hun River

### Historical data availability

Data quality is an important and difficult problem in land-use and land cover change analysis [30], [56], and this is especially true when using historical information that dates back well before remotely sensed data were available. The historical data, especially historical maps, generally carry a higher degree of positional inaccuracy and uncertainty through the use of different sources and lack of geographic coordinates [57]. These “inaccuracy” and “uncertainty” can affect the results of land use change and landscape metrics calculation. Several standardizing historical maps methods have been proposed [58]–[60]. Different from land use change studies with historic maps [13], [34], [60], this research focused on urban landscape change. So, we chose old urban administrative maps, which were acquirable and relatively accurate. The remote sensing data was first geographically corrected, and the historical data were geographically corrected using remote sensing data based on unchanged significant landmarks, such as Shenyang Imperial Palace, Fuling tomb, Zhaoling tomb and unchanged roads around them. Although the historical data contain errors, the data are for the most part meaningful in terms of the urbanization process and the city's historical trajectory.

Because of data limitations, we were unable to study the internal changes in urban development. Major internal changes have occurred in Shenyang through urban renewal and relocation of houses, which have taken place since 2000. The Tiexi district of Shenyang, which was a heavy industrial district before 2000, has been completely transformed into a residential district. Internal change and the building height increase are important parts of urbanization studies, and it will be emphasized in our future research.

### Social and environmental problems

The rapid growth of urban area brought a series of social and environmental problems both in local and regional regions. The area of urban expansion mainly transformed farmlands, which caused the loss of farmland. Population increase and industry development caused the increase in energy and water consumption, which caused the environmental problems including: groundwater funnel area expansion, surface and ground water pollution and air pollution, etc. The Liao River watershed, which includes the city of Shenyang, has become one of the most severely polluted area in China in the last several decades. Concentration of population in urban area had caused social problems, such as the traffic jam and the shortage of public resources, common issues in huge cities.
